# Miniaturized On-Ground 2.4 GHz IoT LTCC Chip Antenna and Its Positioning on a Ground Plane

**DOI:** 10.3390/s23063007

**Published:** 2023-03-10

**Authors:** Jaime Molins-Benlliure, Marta Cabedo-Fabrés, Eva Antonino-Daviu, Miguel Ferrando-Bataller

**Affiliations:** Antennas and Propagation Lab (APL), Instituto de Telecomunicaciones y Aplicaciones Multimedia (iTEAM), Universitat Politècnica de València (UPV), C/ Camí de Vera s/n, 46022 Valencia, Spain; jaimoben@iteam.upv.es (J.M.-B.); marcafab@dcom.upv.es (M.C.-F.); evanda@upvnet.upv.es (E.A.-D.)

**Keywords:** chip antenna, LTCC, small antennas, IoT antenna, bluetooth, Wi-Fi, 2.4 GHz

## Abstract

This paper presents a very low-profile on-ground chip antenna with a total volume of 0.075$\lambda_{0}$× 0.056$\lambda_{0}$× 0.019$\lambda_{0}$ (at f${}_{0}$ = 2.4 GHz). The proposed design is a corrugated (accordion-like) planar inverted F antenna (PIFA) embedded in low-loss glass ceramic material (DuPont GreenTape 9k7 with $\epsilon_{r}$ = 7.1 and tanδ = 0.0009) fabricated with LTCC technology. The antenna does not require a clearance area on the ground plane where the antenna is located, and it is proposed for 2.4 GHz IoT applications for extreme size-limited devices. It shows a 25 MHz impedance bandwidth (for S${}_{11}$ < −6 dB), which means a relative bandwidth of 1%). A study in terms of matching and total efficiency is performed for several size ground planes with the antenna installed at different positions. The use of characteristic modes analysis (CMA) and the correlation between modal and total radiated fields is performed to demonstrate the optimum position of the antenna. Results show high-frequency stability and a total efficiency difference of up to 5.3 dB if the antenna is not placed at the optimum position.

## 1. Introduction

Internet of Things (IoT) introduces a new scenario comprising all connected devices that require RF capabilities for their connection at 2.4 GHz ISM band for WLAN/Wi-Fi/Sensors (Bluetooth, Zigbee, RFID, and NFC) applications. The 2.4–2.48 GHz band is widely used in machine-to-machine (M2M) communications, and often, the wavelength at that frequency ($\lambda_{0}$ = 125 mm) poses a challenge for designing resonating antennas to be installed in size-limited devices. In the past decade, the interest in miniaturizing antennas [1] increased as a consequence of the inclusion of antennas in all kinds of IoT devices, even in ones with extremely limited space which required highly miniaturized antennas. These reduced-size antennas are widely known as small antennas (ESAs), and by definition, they satisfy ka<1 (where *k* is the wavenumber and *a* is the smallest radius of a sphere containing the antenna). Miniaturization techniques enable the installation of antennas in such constrained scenarios, but in general, reducing the size of an antenna leads to a reduction in bandwidth and efficiency, and compromises its radiation properties. The Chu/Wheeler limit [2,3] is widely used to evaluate the radiation limitations of ESAs in terms of impedance bandwidth and efficiency. Miniaturization increases the Q-factor [4,5,6,7] and the sensitivity to the scenario where the antennas are installed [8]. It is important to note that reducing the antenna volume also reduces its bandwidth and efficiency.

Multiple single-band miniaturized antenna solutions have been presented for the 2.4 GHz ISM band for WLAN/Wi-Fi/Sensors applications. A folded strip and a slot are combined in [9] in a compact FR4 board, resulting in an antenna with a size of 0.073 × 0.052 × 0.008$\lambda_{0}^{3}$ and obtaining a bandwidth of 5.09% for WLAN applications. In addition, a planar quasi-isotropic antenna with a folded dipole, two loaded loops, and a coplanar stripline on a PCB is analyzed in [10], obtaining an antenna with a total size of 0.165 × 0.164× 0.006$\lambda_{0}^{3}$ and an impedance bandwidth of 0.99%. Furthermore, a modified meander line microstrip patch antenna is presented in [11] with a total size of 40 × 10 × 1.6 mm${}^{3}$ and a 12.5% impedance bandwidth for IoT applications. Lastly, a compact microstrip filter antenna is proposed in [12] for ISM band and 4G applications with a 45 × 42× 0.81 mm${}^{3}$ volume and a 50% impedance bandwidth.

Regarding dual-band solutions [13,14,15,16], several antennas have been proposed to cover two ISM/Wi-Fi/WLAN bands. In [13], a textile PIFA antenna is analyzed with a 140 × 80 × 6 mm${}^{3}$ size covering the 433 MHZ and 2.4 GHz ISM bands. A reconfigurable FR4 microstrip-based solution is presented in [14], covering the 2.4 GHz and 2.8 GHz ISM bands for IoT applications. Yet, another dual-band solution is proposed in [15] for ISM/Wi-Fi/WLAN applications with a coplanar waveguide antenna working at the 2.45 GHz and 5.65 GHz bands with a total size of 23 × 23× 0.79 mm${}^{3}$. Triple-band solutions [17,18] have been also investigated. In [17], a conformal and electronically reconfigurable antenna is presented for portable devices covering the 2.45 GHz (ISM, Wi-Fi, and WLAN), 3.3/3.5/3.9 GHz (WiMAX), and 4.1/4.9 GHz (4G/5G) bands with the use of a modified triangular patch radiator, two open-ended stubs, and PIN diodes with a total size of 30 × 25× 0.254 mm${}^{3}$. In addition, in [18], a miniaturized antenna based on a square split-ring resonator that operates at the 2.4 GHz, 3.7 GHz, and 5.8 GHz WLAN/WiMAX is proposed, with a volume of 33 × 22 × 1.6 mm${}^{3}$.

Antennas for wearable applications are receiving more attention due to the increasing presence of wireless devices for health and sports tracking, including flexible [19,20] and textile [21] based solutions. Two solutions for earphone integration are presented in [22,23]: one with a chip antenna and another with a wideband loop antenna. Additionally, a planar solution on a semiflexible substrate is proposed in [24], featuring an I-shaped monopole and an inverted L-shaped slit with a size of 0.016 × 0.1 × 0.004$\lambda_{0}^{3}$ and a 5.7% bandwidth.

Reconfigurable antennas [17,25,26,27] are also used in 2.4 GHz IoT applications. In [25], a planar complementary reconfigurable antenna with the combination of electric/magnetic dipoles is analyzed with a size of 0.285 × 0.31 × 0.065$\lambda_{0}^{3}$, and in [26], a reconfigurable microstrip antenna with a slot and a ground plane modification is studied obtaining an impedance bandwidth of 8.7% and a size of 0.016 × 0.016 × 0.007$\lambda_{0}^{3}$. In addition, a 25 × 25 × 1.6 mm${}^{3}$ five-band reconfigurable patch antenna is presented in [27] for WLAN/WiMAX applications.

Miniaturized implantable antennas are necessary for biomedical applications [28,29]. Several solutions have been proposed recently. Another application that is gaining more attention is RFID with the miniaturization of RFID tag antennas [30,31,32,33]. RFID tag antennas are used in many devices to identify tagged objects.

In the early 2000s, there was a surge of interest in miniaturized antennas embedded in ceramic substrates to address the size limitations of traditional solutions such as monopoles and PIFAs. These antennas are commonly known as chip antennas [34,35,36,37,38,39] and have since become primarily commercial solutions due to their low cost when produced in mass quantities. However, there are also low-cost non-ceramic options available, such as the multilayer PCB antenna analyzed in [40], with a size of 0.026 × 0.013 × 0.007$\lambda_{0}^{3}$. It is worth noting that while there were many miniaturized antennas proposed in the past, chip antennas have become the predominant solution in the market today.

The use of Low-Temperature Cofired Ceramic (LTCC) technology is prevalent in the manufacturing of chip antennas. LTCC technology involves stacking ceramic layers with high electric permittivity and low loss tangent, along with metallic sheets and vias with high precision. This technology allows for a high degree of design freedom in creating multilayer antennas, owing to the availability of layers with different thicknesses. However, strict guidelines must be followed during the manufacturing process, such as maintaining proper via separation, adhering to stacking limitations, and accounting for the shrinkage factor that occurs after placing the layers in the oven.

Chip antenna manufacturers provide guidelines for appropriate antenna placement in different scenarios. This is because the contribution of the connected ground plane to small antennas is crucial, and small antennas are particularly sensitive to their surrounding environment. Studies have shown that a significant percentage of the total radiated power of an antenna is produced by the ground plane [41]. Therefore, careful attention must be paid to the size and shape of the ground plane, as well as the placement of the antenna [42,43], in order to avoid degradation of the radiation properties of the device.

The Theory of Characteristic Modes (TCM) [44,45] is widely used for the analysis of metallic structures. Characteristic modes correspond to natural resonances of the structure and provide a great advance in getting information about how to properly feed the structure to excite the desired characteristic modes and enhance the radiation properties. As stated above, the radiation properties of ground planes play a really important role in the radiated fields of a small antenna. Investing effort to analyze ground plane modes [46,47,48,49,50] provides suitable information to place the small antenna properly and, in consequence, excite the desired ground plane modes enhancing the radiation properties of a device.

The Characteristic modes analysis (CMA) of ground planes with different geometries is well documented in the literature, and it is straightforwardly calculated in several electromagnetic simulators. On the contrary, when the antenna is placed on the ground plane, the analysis becomes more complex. When the antenna has a narrowband resonant behavior and a complex structure, such as the proposed antenna, fine meshing is required, which leads to time-demanding simulations and the emergence of issues such as crossing avoidance and modal tracking. In addition, the theoretical background of TCM is well established for lossless structures; hence, dielectric materials can not be easily included. To address this issue, in this paper, we only use CMA to analyze the connected ground plane and then correlate the modal radiated field of each mode to the total radiated field of the antenna+ground plane. With this strategy, high-complexity modal analysis is avoided, and the correlation of modal and total fields is used to approximate which modes of the ground plane are excited. The electromagnetic simulator utilized for the CMA is Feko software.

All the cited antennas require a clearance area (area free of metal dedicated to the antenna) on the PCB or substrate where they are installed. Some of them show a low radiation efficiency and narrow bandwidth due to their reduced size, which is, however, sufficient for the high sensitivity (<−100 dBm) that some receivers exhibit for IoT applications. The proposed LTCC antenna (designed in the CST Studio simulator) does not require a clearance area, and in Table 1, antennas with similar features are summarized. All of them show a higher impedance bandwidth, but the proposed antenna is unique in clearance area requirements (clearance-free), and it can be placed straight away on a ground plane and be operative with limited bandwidth and radiation efficiency. In addition, its resonance frequency remains stable independent of the ground plane size. The proposed antenna is highly suitable for installation in extremely size-limited devices which can not provide a dedicated area for the antenna installation.

Furthermore, manufacturers of chip antennas provide guidelines for placing the antenna on the optimum position of a PCB, but only in general, without a clear understanding of the physical mechanisms involved. In this paper, the proposed antenna is used to analyze the degradation of the radiating performance of the device (antenna+ground plane) depending on the size of the ground plane and the position of the antenna using the CMA and the correlation of total and modal radiation patterns to demonstrate which is the optimum position of the antenna. The paper is organized as follows: Section 2 describes the LTCC antenna geometry, and in Section 3, the analysis in terms of matching and total efficiency depending on the position of the antenna on ground planes of different sizes is performed. Characteristic modes analysis of the ground plane and correlation between total and modal fields are used to support the findings. Finally, in Section 4, the conclusions are presented.

## 2. Proposed LTCC Chip Antenna

**Figure 1:**
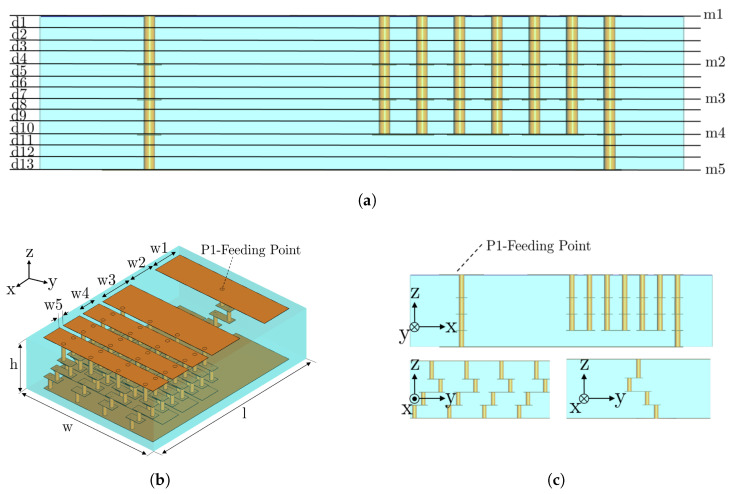
Geometry of the proposed low-profile LTCC chip antenna. Blue color represents the ceramic substrate, and orange color is the metallic layers and vias. (**a**) Side view with all LTCC layers specified, (**b**) overall view of the antenna (dimensions in Table 2), and (**c**) side views of the antenna.

The proposed miniaturized design is a corrugated (accordion-like) planar inverted F antenna (PIFA) embedded in a ceramic substrate and fabricated with LTCC technology, with a total size of 9.3 × 7 × 2.45 mm${}^{3}$ (0.075 × 0.056 × 0.019$\lambda_{0}^{3}$). The antenna geometry is depicted in Figure 1, and its dimensions are detailed in Table 2. The antenna is meant to be installed on a metallic ground plane with no clearance area requirement. Due to its reduced size and the absence of clearance, the bandwidth (25 MHz for S${}_{11}$ < −6 dB, which means a relative bandwidth of 1%) is compromised. However, it shows large frequency stability when the antenna is displaced on different-sized planes.

LTCC fabrication consists of stacking glass–ceramic sheet layers, including metallic sheets, vias, and components in between. The proposed antenna is fabricated using DuPont GreenTape 9k7 (DuPont (U.K.) Ltd., Bristol, UK) ($\epsilon_{r}$ = 7.1, tanδ = 0.0009) low-loss glass ceramic dielectric tape, silver sheets, and silver vias. Figure 1a describes the 13 stacked ceramics layers (d1–d13) with 112 μm or 224 μm thickness. Figure 1b,c show the overall view and side views of the antenna with the grooves/ridges creating the accordion shape, respectively.

**Figure 2:**
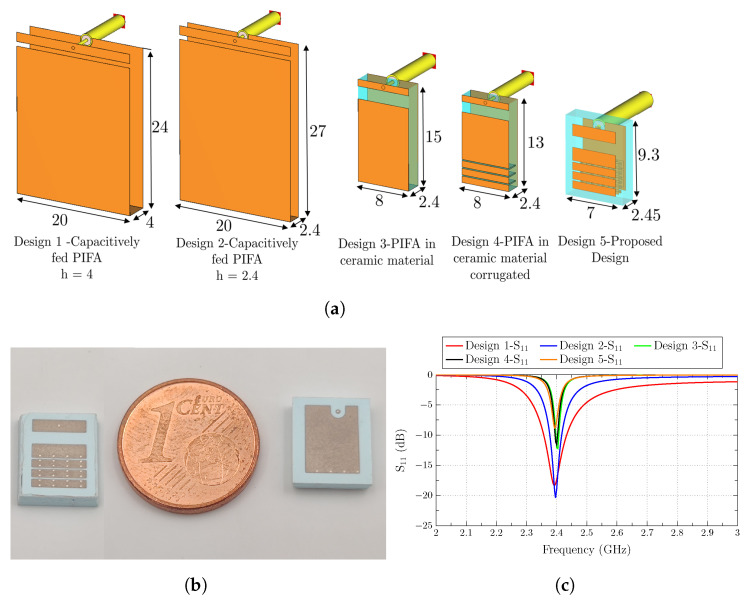
(**a**) Miniaturization process for obtaining the proposed antenna (from Design 1 to the proposed design (Design 5)) with dimensions in mm, (**b**) front and bottom view of the fabricated antenna, and (**c**) reflection coefficients (S${}_{11}$) from Design 1 to Design 5.

Miniaturization is obtained with the use of a high permittivity ceramic substrate and the inclusion of four ridges and three grooves to increase the electric length of the antenna and reduce its size. The design process for the proposed antenna is depicted in Figure 2a.

The first analyzed antenna is a capacitively fed PIFA of 4 mm height (Design 1 of Figure 2a). PIFA antennas are low-profile, and they can be placed on a ground plane without the need for a clearance area. In addition, the fact of feeding the antenna at the furthest point from the short circuit simplifies the future fabrication of a chip antenna, which will be welded to a PCB. The first size reduction applied to the PIFA antenna is its height reduction to 2.4 mm (Design 2 of Figure 2a), which comes with a reduction in impedance bandwidth (see Figure 2c). The following step to compact the low-profile PIFA is to embed the antenna in a ceramic substrate (Design 3 of Figure 2a). As observed, the size of the PIFA antenna is considerably reduced thanks to the use of a high-permittivity substrate. In this case, although the resonance frequency is preserved, the impedance bandwidth is decreased.

To further reduce the size of Design 3, the antenna is corrugated to increase its electrical length (Design 4 of Figure 2a), and lastly, in Design 5 (final design), the vertical walls created by the corrugation of Design 3 are replaced by vias, creating an additional reactive effect which shifts the resonance to lower frequencies and permits the extremely reduced size of the proposed design. The inclusion of so many vias in such a limited space spurs the use of LTCC technology to fabricate the antenna. The last design accounts for the restrictions of the LTCC fabrication process, where the vias must be meandered (see Figure 2b,c) because no more than four layers with vias at the same point can be stacked. Otherwise, bulge effects may appear.

In Figure 2c, it can be observed that the impedance bandwidth decreases with the antenna miniaturization, mainly when the antenna height is reduced and when a high-permittivity substrate is used. The height reduction produces a decrease in ℜ(Z${}_{11}$) close to 0 Ω, except a sudden high-impedance peak at the frequency where the antenna anti-resonates. The imaginary part also shows a high slope change at the anti-resonance frequency. This behavior produces a decrease in the impedance bandwidth when the antenna height is reduced. In addition, the high-permittivity substrate permits the size reduction in the antenna, but increases its stored energy (Q-factor) and decreases its impedance bandwidth. The extreme miniaturization and the low-profile characteristics of the antenna, which can be placed straightforwardly on a ground plane with no clearance area, produce the narrow bandwidth and low-efficiency features of the antenna.

A really compact chip antenna is then obtained with clearance-free capabilities, showing a 9.3 × 7 × 2.45 mm${}^{3}$ (0.075 × 0.056 × 0.019$\lambda_{0}^{3}$) size. In Figure 2b, the front and back sides of the fabricated antenna are depicted next to a 1 Euro cent coin in order to show its reduced size.

## 3. Ground Plane Study

In this section, the radiation properties of the antenna in terms of matching and total efficiency are studied depending on the location of the proposed design at different-sized ground planes. The analysis quantifies the degradation of the radiation properties when an antenna is not placed in a proper place.

As mentioned in the introduction, the total radiated power of a small antenna is greatly affected by the environment surrounding the antenna. In addition, a great percentage of the total radiated power is produced by the induced currents on the ground plane where the antenna is placed. This is well known by the small antenna manufacturers, and generally, they provide guidelines for their antenna products’ installation. In this paper, we provide both a theoretical and experimental study based on a characteristic modes analysis (CMA) to obtain which modes of the ground plane are excited.

**Figure 3:**
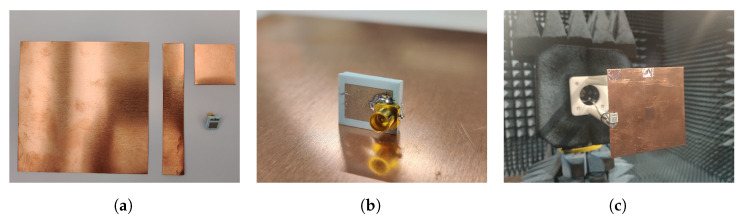
Pictures of the (**a**) analyzed ground planes manufactured with copper sheets and the fabricated antenna, (**b**) antenna with connector, (**c**) measurement set-up in the anechoic chamber.

Three ground plane sizes are studied: an electrically large (a = b = 100 mm >λ/2), an electrically small (a = b = 30 mm <λ/4), and a thin (a = 100 mm and b = 20 mm) ground plane. In Figure 3a, the three copper ground planes with the proposed antenna used for the experimental analysis are depicted. In addition, the connectorized antenna is depicted in Figure 3b inserted in different ground plane positions where different holes were drilled to connect the antenna. In Figure 3c, the set-up to measure the radiation patterns in the anechoic chamber is depicted.

### 3.1. Characteristic Modes Analysis (CMA)

The Theory of Characteristic Modes (TCM) [44,45] decomposes the total current of an arbitrary structure in a set of currents (modes) with orthogonal radiation properties. The modal subdivision provides visual and suitable information to properly excite the structure. Furthermore, it helps with the understanding of the radiation mechanism of the analyzed antenna. Characteristic modes are obtained from the generalized impedance matrix of a structure [*Z*], which is calculated with the method of moments (MoM), and then characteristic modes or eigenvectors (J${}_{n}$) and eigenvalues ($\lambda_{n}$) are obtained with the following equation:

(1) $$\left[X\right]\vec{J_{n}}=\lambda_{n}\left[R\right]\vec{J_{n}}$$

where [*X*] and [*R*] are the imaginary and real parts of the generalized impedance matrix of the structure [*Z*], respectively.

Eigenvalues ($\lambda_{n}$) are frequency-dependent and provide information about the radiation properties of the associated current mode (J${}_{n}$). The mode is considered capacitive and stores electric energy when $\lambda_{n}$ is negative, and inductive and stores magnetic energy when $\lambda_{n}$ is positive. When $\lambda_{n}$ = 0, the mode is at resonance. Different modal attributes for the physical interpretation of the eigenvalues have been proposed [52], such as the modal significance (MS), the variation with frequency of the Eigenvalues, the characteristic angle, or the modal quality factor. In this paper, the characteristic angle ($\alpha_{n}$) is used for the analysis, and it is defined by the following:

(2) $$\alpha_{n}=180^{\circ }-\tan^{-1}\left(\lambda_{n}\right)$$

When the mode is at resonance, $\lambda_{n}=0$ and $\alpha_{n}=180^{\circ }$.

The CMA of different square plates is well detailed in the literature. The decomposition of the total current into modal currents or characteristic modes (J${}_{n}$) provides a visual perspective of the radiation mechanism of the analyzed plate. It helps to determine which modes are good candidates to be excited and, consequently, which is the optimum position of the feeding. However, the CMA of the ground plane, including the small antenna, requires an extremely detailed meshing which is accompanied by time-demanding simulations, along with modal tracking and crossing avoidance issues. Furthermore, the antenna includes a lossy ceramic substrate, which complicates the analysis because the CMA is only well established for lossless structures, and commercial simulators do not analyze any lossy substrate. To face this problem, we analyze the isolated ground plane with the CMA, and then we correlate the radiated fields of each mode with the total radiated fields of the ground plane, including the chip antenna, at different positions. The correlation is then used as a metric to quantify approximately which mode of the ground plane is excited.

The correlation is detailed in (3), where $g_{n}$ is the radiation pattern associated with mode J${}_{n}$ of the analyzed ground plane, and $g_{T(x,y)}$ is the total radiation pattern of the antenna + ground plane when the antenna is placed at (x,y). The superscript ${}^{H}$ denotes Hermitian, and Ω=(θ,ϕ) is the solid angle.

(3) $$\rho_{n,T(x,y)}=\frac{\underset{4\pi }{\;\int \int \;}g_{n}^{H}\left(\Omega \right)g_{T(x,y)}\left(\Omega \right)\;d\Omega }{\sqrt{\underset{4\pi }{\;\int \int \;}g_{n}^{H}\left(\Omega \right)g_{n}\left(\Omega \right)\;d\Omega \cdot \underset{4\pi }{\;\int \int \;}g_{T(x,y)}^{H}\left(\Omega \right)g_{T(x,y)}\left(\Omega \right)\;d\Omega }}$$

Thanks to the correlation, we can analytically approximate which modes are excited. For a proper mode excitation, the antenna must be placed in a current maximum, and the currents of the antenna must flow in the same direction as the modal currents. These two requirements are critical for the proper excitation of any mode and for the enhancement of the radiation properties of the system.

### 3.2. Large Ground Plane (a = b = 100 mm)

**Figure 4:**
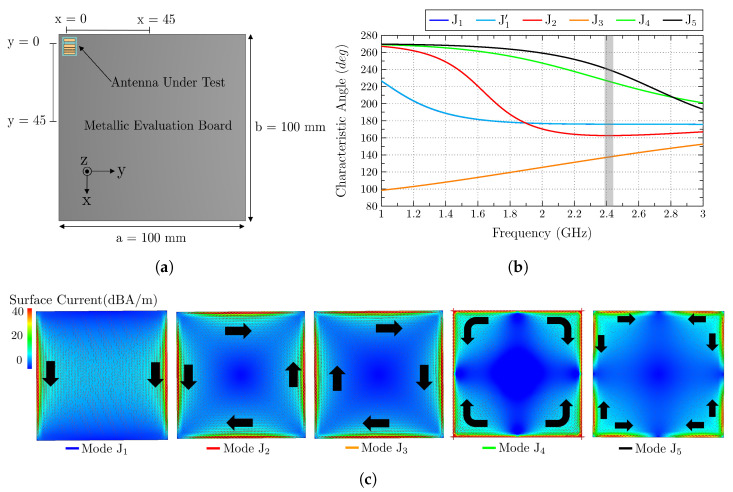
(**a**) Analyzed ground plane of 100 × 100 mm${}^{2}$ with the antenna at four different positions, (**b**) characteristic angles of the isolated ground plane modes (J${}_{1}$–J${}_{5}$) with the operating frequency band colored in gray, and (**c**) current distribution of the isolated ground plane modes (J${}_{1}$–J${}_{5}$). J${}_{1}^{\prime }$ is equivalent to J${}_{1}$, but rotated 90${}^{\circ }$ (degenerated mode). Black arrows describe the direction of the currents.

The first analysis consists of an electrically large square ground plane with dimensions a = b = 100 mm, which correspond to a >λ/2. For this study, four positions of the antenna are considered, top-left corner P1(x,y) = (0,0), top-middle P2(x,y) = (45,0), left-middle P3(x,y) = (0,45), and center P4(x,y) = (45,45). In Figure 4a, the evaluation set-up is detailed.

The initial step focuses on the CMA of the isolated square ground plane. In Figure 4b,c, the characteristic angle and the current distribution of each mode are depicted, respectively. The gray bar represented in Figure 4b indicates the band where the proposed antenna is working. At that band, modes J${}_{1}$/J${}_{1}^{\prime }$, which are the fundamental modes (vertical/horizontal modes), have already resonated and have values close to 180${}^{\circ }$, and they have the potential to be excited. Mode J${}_{2}$ also resonates before the operating band and also has characteristic angle values close to 180${}^{\circ }$. This is a mode with four nulls at the corners of the plane and a maximum in the middle of the edges, and it is also a potential candidate to be excited. Mode J${}_{3}$ is considered the loop mode, which does not resonate. Mode J${}_{4}$ and J${}_{5}$ resonate at higher frequencies, but they may be excited because they are not far from resonance, and the capacitive behavior that they show at the operating band can be compensated. Mode J${}_{4}$ has four current nulls in the middle of the four edges, and mode J${}_{5}$ has the same four nulls, along with four nulls at the corners. Since the ground plane is considered electrically big, all the mentioned modes can be potentially considered to be excited due to their relative proximity to resonance.

**Figure 5:**
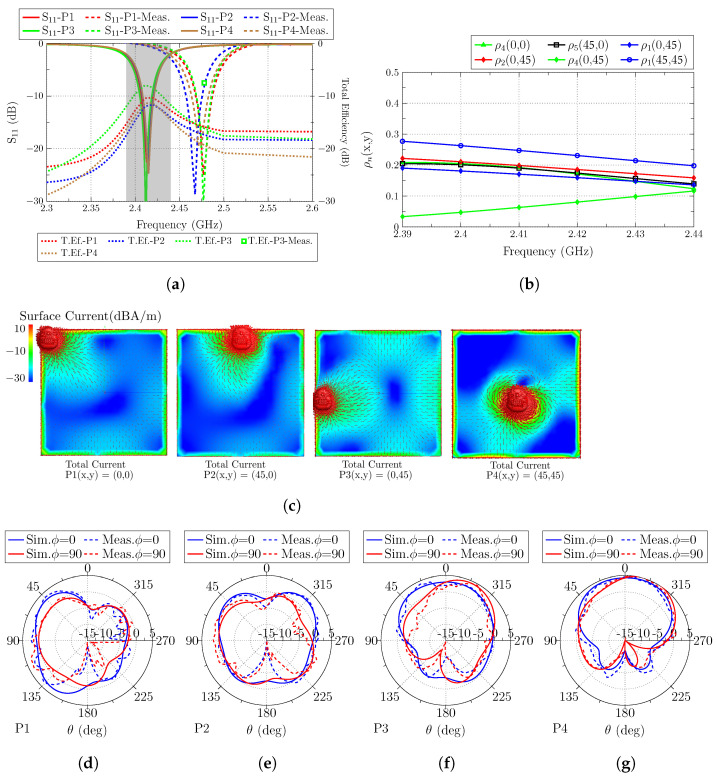
(**a**) Reflection coefficient (S${}_{11}$) and total efficiency (dB) of the antenna located at the four analyzed positions (the frequency band colored in gray corresponds to the operating band analyzed with the correlation), (**b**) correlation (detailed in Equation (3)) of total and modal fields, (**c**) total current distribution of the antenna at the four positions, and radiation patterns at resonance when the antenna is placed at (**d**) P1(x,y) = (0,0), (**e**) P2(x,y) = (45,0), (**f**) P3(x,y) = (0,45), and (**g**) P4(x,y) = (45,45).

Once the ground plane is analyzed, the antenna is placed at the four analyzed positions, and the reflection coefficient (S${}_{11}$) and the total efficiency are calculated and depicted together in Figure 5a. The result for the S${}_{11}$ parameter shows a very narrow bandwidth for the operating band (25 MHz for $S_{11}<-6$ dB), and high stability with no detuning nor bandwidth degradation when the antenna is displaced. The measured S${}_{11}$ results show a slight frequency shift due to fabrication tolerances, but show high stability with the antenna displacement. The total measured efficiency is also measured, with good agreement with the simulated results.

As observed, the total efficiency is greatly affected by the position of the antenna. The highest value of total efficiency (−8.5 dB) is obtained when the antenna is placed at P3(x,y) = (0,45). In Figure 1b, the correlation between the total fields and each modal field is depicted, and in the case of (x,y) = (0,45), modes J${}_{1}$, J${}_{2}$, and J${}_{4}$ are excited. The first two present higher correlation because the antenna is placed at a current maximum of both modes and for mode J${}_{4}$ at a minimum. The second higher value (−10.2 dB) is obtained when the antenna is placed at P1(x,y) = (0,0), where only mode (J${}_{4}$) is properly excited because only mode J${}_{4}$ has a current maximum in the corner of the ground plane. Lastly, for positions P2(x,y) = (45,0) and P4(x,y) = (45,45), the lowest total efficiency value (−12 dB) is obtained. For P2(x,y) = (45,0), only mode J${}_{5}$ is excited and at a low level because it has a current minimum at the excitation point. All the other modes are not excited because the current flow of the antenna is orthogonal to the current flow of all the modal fields. For P4(x,y) = (45,45), the antenna is placed at a position where most of the modes have a null. Only mode J${}_{1}$ can be excited because its current flow has the same direction as the antenna, but the antenna is not placed at the current maximum.

The total current of the ground plane with the antenna placed at the four positions is depicted in Figure 5c for its visual comparison with the modes. The radiation patterns (Directivity) for planes ϕ = 0${}^{\circ }$ and ϕ = 90${}^{\circ }$ are also depicted for all the analyzed positions (Figure 5d–g), and the measured radiation pattern for the best total efficiency position is also added. The measured radiation patterns differ from the simulated ones at θ = 180${}^{\circ }$ for both planes due to the blocking effect of the positioner of the anechoic chamber.

As a first conclusion, the best position for the antenna is the middle-left position P3(x,y) = (0,45) because most of the first analyzed modes of the ground plane, which are close to resonance, exhibit a current maximum in the perimeter of the ground plane and especially in the middle of each side. In addition, the current flow of the antenna has the same direction as the modal currents.

### 3.3. Small Ground Plane (a = b = 30 mm)

**Figure 6:**
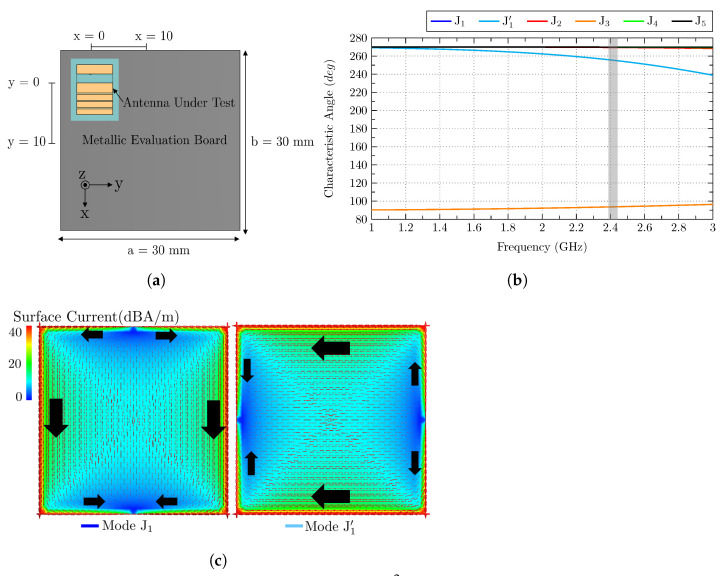
(**a**) Analyzed ground plane of 30 × 30 mm${}^{2}$ with the antenna at four different positions on the ground plane, (**b**) characteristic angles of the isolated ground plane modes (J${}_{1}$–J${}_{5}$) with the operating frequency band colored in gray, and (**c**) current distribution of mode J${}_{1}$ and J${}_{1}^{\prime }$ of the isolated ground plane. Black arrows describe the direction of the currents.

The second subsection analyzes an electrically small square ground plane with the dimensions a = b = 30 mm, which correspond to a <λ/4. As in the previous subsection, the same four positions of the antenna are considered: top-left corner P1(x,y) = (0,0), top-middle P2(x,y) = (12,0), left-middle P3(x,y) = (0,12) and center P4(x,y) = (12,12), detailed in Figure 6a.

The CMA of the isolated plane shows that at the operating band (gray band of Figure 6b), all the modes have not resonated yet due to the limited size of the plane. Only modes J${}_{1}$ and J${}_{1}^{\prime }$ have a lower value of characteristic angle, and they will be the only modes that will be excited. In Figure 6c, the current distribution of modes J${}_{1}$ and J${}_{1}^{\prime }$ at 2.4 GHz is represented. Since the modes are far from resonance, it can be observed that currents are not only flowing vertically or horizontally (like at resonance), but they have an external path beginning in the current minimum (at the middle of one of the edges) and flowing throw the edge of the perimeter (C-shape path) until the middle of the other edge.

**Figure 7:**
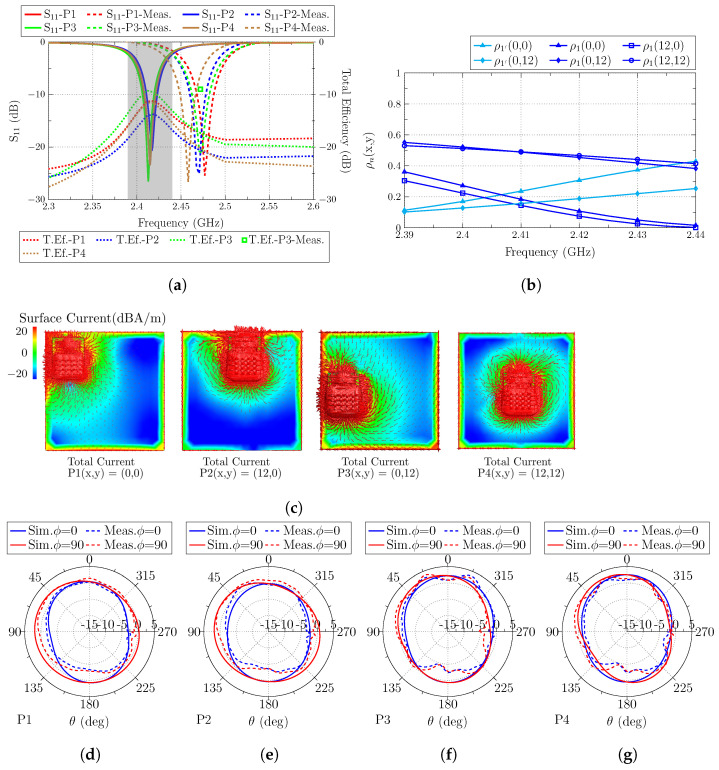
(**a**) Reflection coefficient (S${}_{11}$) and total efficiency (dB) of the antenna located at the four analyzed positions (the frequency band colored in gray corresponds to the operating band analyzed with the correlation), (**b**) correlation (detailed in Equation (3)) of total and modal fields, (**c**) total current distribution of the antenna at the four positions, and radiation patterns at resonance when the antenna is placed at (**d**) P1(x,y) = (0,0), (**e**) P2(x,y) = (12,0), (**f**) P3(x,y) = (0,12), and (**g**) P4(x,y) = (12,12).

After the ground plane analysis, the antenna is analyzed at the four ground plane positions. The reflection coefficient (S${}_{11}$) and the total efficiency are depicted together in Figure 7a. The result for the S${}_{11}$ parameter shows the same narrow bandwidth operating band (25 MHz for $S_{11}<-6$ dB) as in the electrically large ground plane and high stability with no detuning nor bandwidth degradation even when the antenna is displaced in such a limited ground plane.

The total efficiency is also affected by the position of the antenna. With a simple inspection of the two modes of the isolated ground plane J${}_{1}$/J${}_{1}^{\prime }$, it is possible to anticipate which position is more suitable to obtain the highest total efficiency. Position P3(x,y) = (0,12) provides the highest efficiency value (−9.5 dB) because the antenna is placed in the middle of the current maximum associated with mode J${}_{1}$, and also, the current flow of the antenna follows the current flow of the mode. In addition, mode J${}_{1}^{\prime }$ is excited with a lower level because it has a current null in that position, but the prolongation of intense currents in the C-shape distribution from the middle-top to middle-bottom point (which appears at such low frequencies) creates an additional current path in phase with the current flow of the antenna. The following efficiency values are −11.4 dB and −11.5 for positions P1(x,y) = (0,0) and P4(x,y) = (12,12), respectively. For P1(x,y) = (0,0), the antenna is placed not in a current minimum nor a maximum for mode J${}_{1}$ and J${}_{1}^{\prime }$. Both modes are excited at a low level, because the antenna current flow has the same direction as the modes, but it is not placed in any of the current maximums. In the case of P4(x,y) = (12,12), only mode J${}_{1}$ is excited with medium level because even though it has the same current flow as the mode, it is not placed in the exact current maximum (edge). The worst result for the total efficiency (−13.5 dB) is obtained at P2(x,y) = (12,0), with a current minimum for mode J${}_{1}$ (with antenna current flow in phase) and a maximum of J${}_{1}^{\prime }$ (but with the current flow orthogonal to the mode). Only mode J${}_{1}$ is then excited in this case, but with a poor level.

Total currents with the antenna placed at the four positions are depicted in Figure 7c for its visual comparison with the modal currents. The radiation patterns (directivity) for planes ϕ = 0${}^{\circ }$ and ϕ = 90${}^{\circ }$ are also depicted in Figure 7 for all the analyzed positions.

After the second analysis, it can be stated that, again, the best position for the antenna placement is the middle-left position, because the antenna is placed in the current maximum of mode J${}_{1}$, and the current flow of the antenna is parallel to the current flow in the maximum of the mode. The worst position is the top-middle P2(x,y) = (12,0) because either the current flow is orthogonal to the current maximum flow of J${}_{1}^{\prime }$ or, in the case of J${}_{1}$, it is in a current minimum.

### 3.4. Long Ground Plane (a = 15 mm and b = 100 mm)

**Figure 8:**
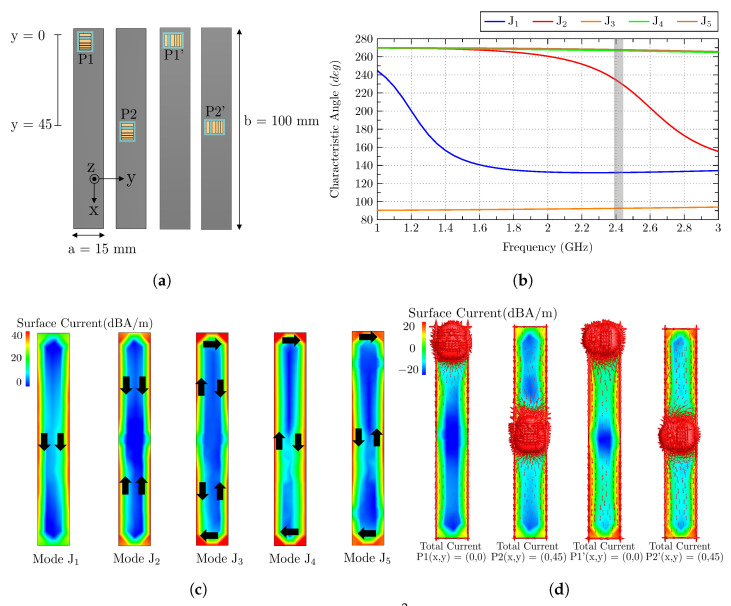
(**a**) Analyzed ground plane of 15 × 100 mm${}^{2}$, with the antenna at two different positions and two polarizations, (**b**) characteristic angles of the isolated ground plane modes (J${}_{1}$–J${}_{5}$) with the operating frequency band colored in gray, (**c**) current distribution of modes J${}_{1}$–J${}_{5}$ of the isolated ground plane (black arrows describe the direction of the currents), and (**d**) total current distribution of the antenna at the four positions.

The last subsection provides the analysis of an electrically long ground plane with dimensions a = 15 mm and b = 100 mm. In this study, due to the reduced width (a) of the ground plane, only two positions are analyzed: Top P1(x,y) = (0,0) and middle P2(x,y) = (0,45). At each position, the antenna is placed in two orthogonal ways (see Figure 8a).

With an inspection of the characteristic angles associated with the modes of the long ground plane (Figure 8b), it can be observed that mode J${}_{1}$ is resonant before the operating band (remarked in gray color), and mode J${}_{2}$ is close to resonance. The other modes resonate at higher frequencies, and by a first impression, it can be stated that only modes J${}_{1}$ and J${}_{2}$ will be present in the analysis. Mode J${}_{1}$ has the current distribution of the vertical mode (see Figure 8c), with nulls at the top and bottom edges and maximums in the middle of the left and right sides. Mode J${}_{2}$ presents a current minimum in the middle of the right/left sides and a current maximum at the top and bottom edges. Mode J${}_{3}$ corresponds to the loop mode, and mode J${}_{4}$ is a higher-order mode, with four current nulls and four maximums. The total currents, including the antenna, are depicted in Figure 8d for a visual comparison between modal and total currents.

**Figure 9:**
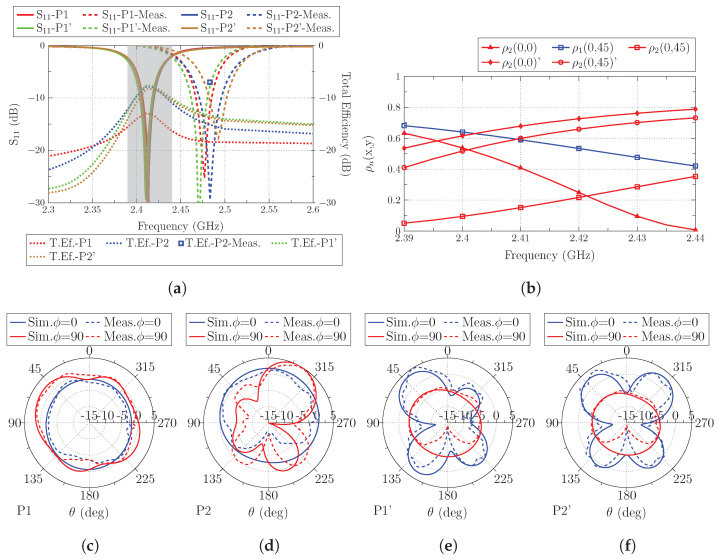
(**a**) Reflection coefficient (S${}_{11}$) and total efficiency (dB) of the antenna located at the four analyzed positions (the frequency band colored in gray corresponds to the operating band analyzed with the correlation), (**b**) Correlation (detailed in Equation (3)) of total and modal fields, and radiation patterns at (**c**) P1(x,y) = (0,0), (**d**) P2(x,y) = (0,45), (**e**) P1’(x,y) = (0,0) and, (**f**) P2’(x,y) = (0,45).

Regarding the matching of the antenna (see Figure 9a), results show the same stability as in previous studies, with no detuning observed when the antenna is displaced, or the polarization is changed.

The total efficiency (Figure 9a) exhibits the highest dynamic range among all the performed studies. On the one hand, the best total efficiency results (−7.6 dB and −7.8 dB) observed in Figure 9a are obtained at positions P1’(x,y) = (0,0) and P2(x,y) = (0,45). By analyzing the correlation (Figure 9b), it can be observed that for P1’(x,y) = (0,0), mode J${}_{2}$ is perfectly excited because the antenna is placed parallel to the top edge, and there is a current maximum flowing in the same direction of the currents of the antenna. For P2(x,y) = (0,45) position, the antenna is clearly exciting mode J${}_{1}$ og the ground plane, because it is placed in the middle of the left and right sides, and since the plane is very thin, both maximums are excited. The next highest value for the total efficiency is obtained for P2’(x,y)= (0,45), where mode J${}_{2}$ is excited but not at the same level, because the antenna is placed in the current minimum. Although it is placed in the maximum for J${}_{1}$, the current flow of the antenna is orthogonal to the currents of this mode. The worst total efficiency value (−12.9 dB) is observed for P1(x,y) = (0,0) positions. The reason for such a low level is that mode J${}_{1}$ has a current minimum and mode J${}_{2}$ has its maximum on the top edge, but it is orthogonal to the current flow of the antenna.

Radiation patterns (directivity) for planes ϕ = 0${}^{\circ }$ and ϕ = 90${}^{\circ }$ are also depicted for all the analyzed positions in Figure 9.

The information gathered in the last analysis shows that for narrow ground planes, the antenna position has a big impact on the total efficiency. Really high correlation and efficiency values are obtained if the antenna is placed in the correct position, always taking into consideration the current distribution of the modes close to resonance and taking also into consideration the current flow of the antenna.

## 4. Conclusions

A low-profile on-ground LTCC chip antenna resonating at 2.41 GHz has been presented. The antenna exhibits a total volume of 0.067 × 0.048 × 0.019$\lambda_{0}^{3}$ and does not require a clearance area on the ground plane where it is installed. Characteristic modes analysis of ground planes with different sizes, and the correlation between the total radiated fields (antenna + ground plane) and modal radiated fields (ground plane) were successfully performed, justifying the best position for the antenna location in terms of total efficiency. This study shows that the antenna is really stable to detuning effects and shows a considerable difference in terms of efficiency from the best to the worst position (5.3 dB difference), as described in Table 3. The best results are obtained when the antenna is placed in the middle of the left side of the ground plane, and the reason is demonstrated by the modal analysis. Due to the size of the antenna and the lack of clearance area requirement, this antenna is a great candidate to be installed in extremely size-limited devices working for 2.4 GHz IoT applications. As further work, once the on-ground (no clearance) chip antenna concept has been presented and the CMA and correlation study to find the optimum location of the antenna in a ground plane has been performed, the impedance bandwidth enhancement of the proposed antenna will be investigated. A comparison between classical chip antennas with clearance area and on-ground chip antennas will be carried out.

## Figures and Tables

**Table 1:** Comparison Table.

Ref.	BW (GHz)	BW (%)	Size (mm${}^{3}$)	Size ($\lambda_{0}^{3}$)	Clearance-Free
[9]	2.362–2.492	5.36%	9.2 × 6.5 × 1	0.073 × 0.052 × 0.008	No
[11]	2.35–2.65	12.5%	40 × 10 × 1.6	0.32 × 0.08 × 0.013	No
[16]	2.4–2.5	4%	30 × 30 × 2	0.24 × 0.24 × 0.016	No
[22]	≈2.4–2.5	≈ 4%	13 × 4.9 × 2	0.1 × 0.04 × 0.016	No
[24]	2.4–2.54	5.7%	19 × 12 × 0.508	0.15 × 0.1 × 0.004	No
[40]	2.4–2.56	7.6%	3.2 × 1.6 × 0.83	0.025 × 0.013 × 0.007	No
[51]	≈2.4–2.45	≈ 2%	7.5 × 4 × 1	0.06 × 0.032 × 0.008	No
Prop.	2.4-2.425	1%	9.3 × 7 × 2.45	0.075 × 0.056 × 0.019	Yes

**Table 2:** Dimensions of the proposed chip antenna (unit: mm).

l	w	h	w1	w2	w3	w4	w5
9.3	7	2.45	1.5	1.5	1.7	1	0.2

**Table 3:** Summary of the results. Red cell: Antenna in a wrong location, Orange cell: Antenna in an intermediate location, Green cell: Antenna in the optimum location.

Ground P. Size	Antenna Position	T.Eff.	Ground P. Size	Antenna Position	T.Eff.
100 × 100 mm${}^{2}$	P1(0,0)	−10.2 dB	100 × 100 mm${}^{2}$	P2(45,0)	−12 dB
100 × 100 mm${}^{2}$	P3(0,45)	−8.5 dB	100 × 100 mm${}^{2}$	P4(45,45)	−12 dB
30 × 30 mm${}^{2}$	P1(0,0)	−11.4 dB	30 × 30 mm${}^{2}$	P2(12,0)	−13.5 dB
30 × 30 mm${}^{2}$	P3(0,12)	−9.5 dB	30 × 30 mm${}^{2}$	P4(12,12)	−11.5 dB
15 × 100 mm${}^{2}$	P1(0,0)	−12.9 dB	15 × 100 mm${}^{2}$	P2(0,45)	−7.8 dB
15 × 100 mm${}^{2}$	P1’(0,0)	−7.6 dB	15 × 100 mm${}^{2}$	P2’(0,45)	−8.3 dB

## Data Availability

Not applicable.
